# Growth kinetics of Cu_6_Sn_5_ intermetallic compound in Cu-liquid Sn interfacial reaction enhanced by electric current

**DOI:** 10.1038/s41598-018-20100-1

**Published:** 2018-01-29

**Authors:** Jiayun Feng, Chunjin Hang, Yanhong Tian, Baolei Liu, Chenxi Wang

**Affiliations:** State Key Laboratory of Advanced Welding and Joining, Harbin Institute of Technology, Harbin, 150001 China

## Abstract

In this paper, electric currents with the densities of 1.0 × 10^2^ A/cm^2^ and 2.0 × 10^2^ A/cm^2^ were imposed to the Cu-liquid Sn interfacial reaction at 260 °C and 300 °C with the bonding times from 15 min to 960 min. Unlike the symmetrical growth following a cubic root dependence on time during reflowing, the Cu_6_Sn_5_ growth enhanced by solid-liquid electromigration followed a linear relationship with time. The elevated electric current density and reaction temperature could greatly accelerate the growth of Cu_6_Sn_5_, and could induce the formation of cellular structures on the surfaces because of the constitutional supercooling effect. A growth kinetics model of Cu_6_Sn_5_ based on Cu concentration gradient was presented, in which the dissolution of cathode was proved to be the controlling step. This model indicates that higher current density, higher temperature and larger joint width were in favor of the dissolution of Cu. Finally, the shear strengths of joints consisted of different intermetallic compound microstructures were evaluated. The results showed that the Cu_6_Sn_5_-based joint could achieve comparable shear strength with Sn-based joint.

## Introduction

With the rapid multi-functionalization and miniaturization of present electronic devices and the popularization of wide band semiconductors like gallium nitride (GaN) and silicon carbide (SiC), the power density and service temperature increase greatly in current integrated circuits^1,2^. The high temperature electrical interconnection materials are in great demand. However, the traditional high melting point (MP) solder requires higher processing temperature in multiple stacking processes, which may damage the circuits and induce many reliability issues^3^.

Recently, Cu_6_Sn_5_, one of the major Cu-Sn intermetallic compounds (IMCs) in the soldering reaction between Cu substrate and Sn-based solder has drawn a lot of attention as one of the alternatives to traditional Sn-based alloys, for its high melting point, better electromigration resistance, better thermal stability and mechanical properties^4–6^, especially for the low cost and simple processing procedure. However, conventional transient liquid phase (TLP) soldering and solid-liquid interdiffusion (SLID) bonding for full-IMCs joint fabrication are rather time consuming, which will induce serious thermal effect on the reliability of packaging systems^7–9^.

Therefore, many attempts have been tried to shorten the bonding time^8^, such as increasing soldering temperature or ultrasonic-assisted bonding^4,5,10^. Recently, a more convenient and effective method of electric current-assisted bonding has been intensively studied, in which solid-liquid electromigration (S-L EM) is utilized to accelerate the bonding process. Huang *et al*. and Hu et al. ^11,12^ have investigated the S-L EM in Cu/Sn-3.5Ag/Cu interconnects, and found that the dissolution rate of the Cu cathode was one order of magnitude higher than that during solid-solid (S-S EM). Liu *et al*.^13^ imposed an electric current density of 1.44 × 10^4^ A/cm^2^ on Cu/Sn/Cu system to fabricate the Cu-Sn interconnections within 180 ms, and achieved a shear strength as high as 67.3 MPa. Ma *et al*.^14^ has investigated the S-L EM under 0.56 × 10^3^ A/cm^2^ in Cu/liquid Sn system by synchrotron radiation real time imaging technology, and found that the Cu_6_Sn_5_ layer at the anode grew linearly with the bonding time. Huang *et al*.^15^ has investigated the size effect on the interfacial reaction, and proposed a concentration gradient controlled (CGC) kinetic model to explain it. However, there are no systematic theories explaining the growth kinetics of Cu_6_Sn_5_ in small-size joints under S-L EM system concerning the size effect. Therefore, it is critical to investigate and understand the S-L EM process integrally from the dissolution of Cu substrate to the diffusion of Cu atoms in molten Sn, and to the deposition of Cu_6_Sn_5_.

This study focus on the morphology evolution and growth kinetics of Cu_6_Sn_5_ in Cu/liquid Sn/Cu system under different electric current densities and temperatures, and takes the Cu concentration gradient into consideration to explain the size effect on the S-L EM system. Furthermore, the correlations between microstructure and shear strength of the Cu-Sn interconnects were studied.

## Results

### Cross-sectional microstructure evolution

**Figure 1:**
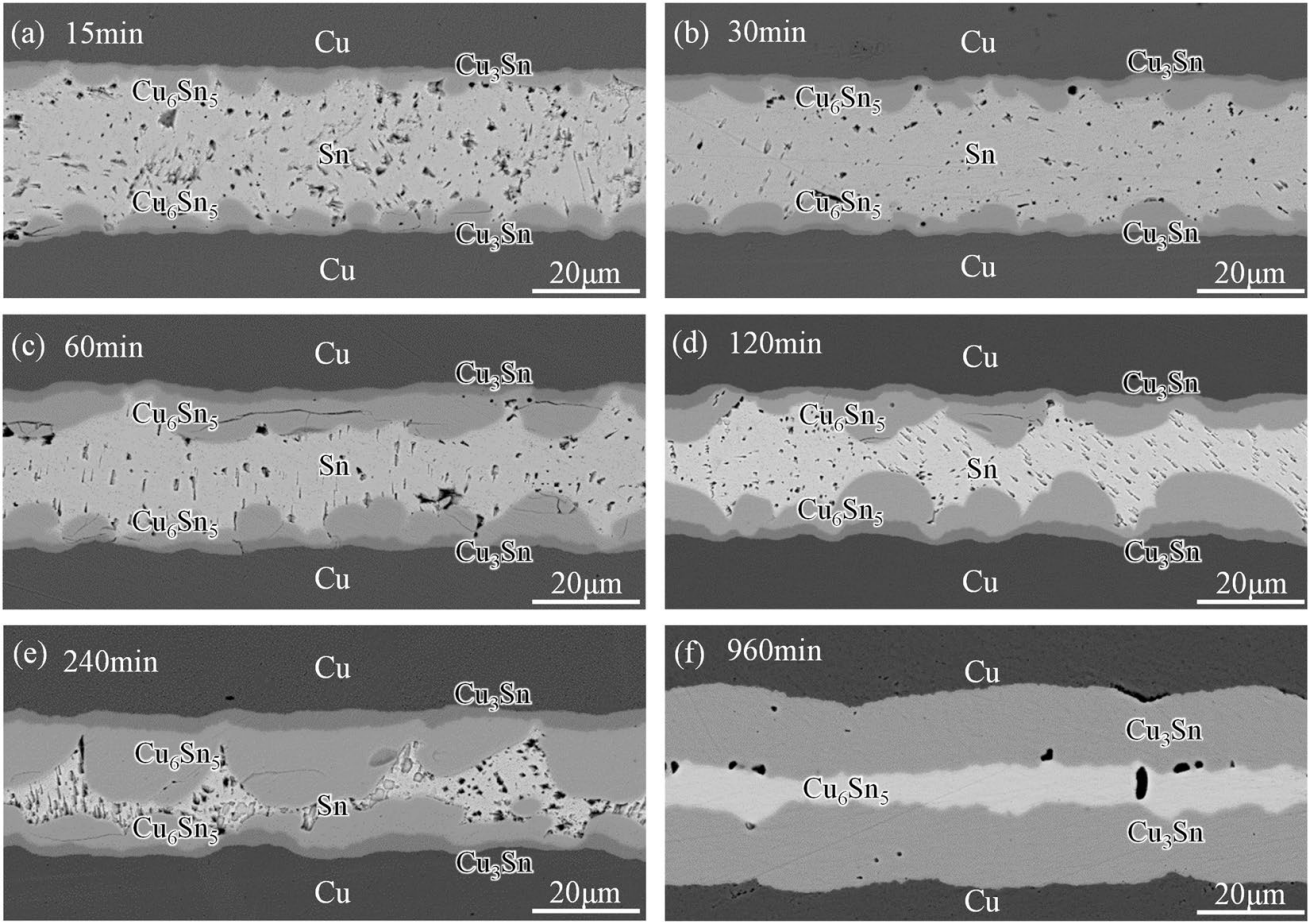
Morphology evolution in Cu-Sn intermetallic joints at 260 °C for various times: (**a**) 15 min, (**b**) 30 min, (**c**) 60 min, (**d**) 120 min, (**e**) 240 min, (**f**) 960 min.

Figure 1 shows the cross-sectional microstructure evolution of interfacial IMCs in solid-liquid Cu-Sn reaction (without electric current) for various times at 260 °C. Two adjacent thin layers of η-Cu_6_Sn_5_ and ε-Cu_3_Sn formed at both Cu/Sn interfaces^7^. The Cu_6_Sn_5_ scallops coarsened continually with bonding time, and the number of scallops decreased accordingly, as shown in Fig. 1a–d. The scallop-type morphology indicates its growth was supply-controlled ripening process, in which Cu_6_Sn_5_ grains grew at the expense of their nearest neighbors. At 240 min (Fig. 1e), the relatively larger Cu_6_Sn_5_ grains grew from the two original boundary planes were about to contact with each other. The residual Sn gradually ran out and the joint then transformed into full IMC joint, as shown in Fig. 1f. After that, Cu_3_Sn would keep growing at the expense of Cu_6_Sn_5_. The Cu_6_Sn_5_ phase still remained in the joint even after 960 min, as shown in Fig. 1f.

**Figure 2:**
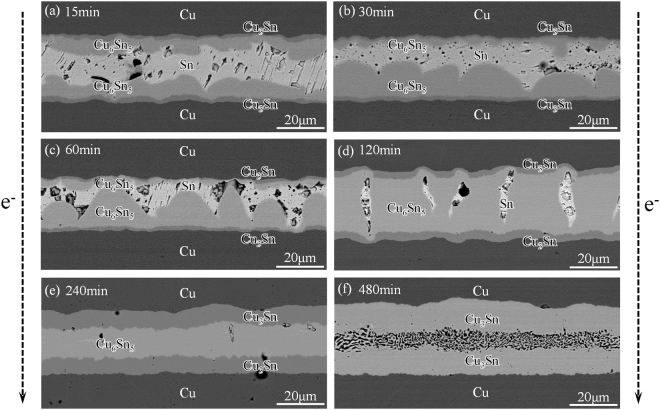
Morphology evolution in Cu-Sn intermetallic joints under a current density of 1.0 × 10^2^ A/cm^2^ at 260 °C for various times: (**a**) 15 min, (**b**) 30 min, (**c**) 60 min, (**d**) 120 min, (**e**) 240 min, (**f**) 480 min.

Figure 2 shows different microstructures of Cu-Sn IMCs under a current density of 1.0 × 10^2^ A/cm^2^ at 260 °C, where the dotted arrow marked by “e^−^” indicates the electron flow direction. The anode Cu_6_Sn_5_ grew more rapidly, whereas the growth of cathode Cu_6_Sn_5_ was suppressed, showing an obvious polarity effect in 30 min (Fig. 2a,b). After 60 min (Fig. 2c), the tip of anode Cu_6_Sn_5_ grains touched the cathode Cu_6_Sn_5_ and merged into larger grains. Afterwards, the residual Sn was gradually isolated by Cu-Sn IMCs and the full-Cu_6_Sn_5_ joint formed after 120 min (Fig. 2d). After the complete consumption of Sn, the S-L EM transformed into S-S EM, and the dominant reaction within the joints became the formation of Cu_3_Sn at the expense of Cu_6_Sn_5_ (Fig. 2e). Cu was the dominant diffusing species, and the Cu atoms slowly diffused through the Cu_3_Sn layer and reacted with Cu_6_Sn_5_. In this stage, the Cu_3_Sn layer at either side grew symmetrically in thickness, showing limited sensitivity to electron current. After 480 min, the Cu_6_Sn_5_ phase fully transformed to Cu_3_Sn, leaving a honeycomb structure in between. The density of Cu_6_Sn_5_, Cu and Cu_3_Sn were 8.28, 8.93 and 8.90 g·cm^−3^, respectively^16^. After calculations, we can find that the volume shrank about 4.39% after the Cu_6_Sn_5_ transformed to Cu_3_Sn. That may be the main reason why the voids formed within the joints. These voids will disappear eventually after long-time reaction.

The EPMA (Electron probe micro-analyzer) analysis was performed to the 15 min sample, and the Cu concentration distribution along the joint was measured (Supplementary Fig. S1). The average Cu concentration dissolved in Sn was 6.86 ± 2.29 at.%, which was higher than the equilibrium Cu concentration at 260 °C shown in phase diagram(~2.401 at.%). Although some details of Cu gradient may be missing during solidification, this result can at least prove that the Cu was oversaturated in liquid Sn.

Another two groups of experiments were performed to further investigate the microstructure evolution under higher electric current density and temperature. When the electric current density increased to 2.0 × 10^2^ A/cm^2^ at 260 °C, as shown in Supplementary Fig. S2, the precipitation rate of anode Cu_6_Sn_5_ increased accordingly, while the growth rate of Cu_3_Sn had no significant change. One difference in morphology was that there were some cellular structures appearing on the anode Cu_6_Sn_5_ grain surfaces, as shown in Fig. S2a and b. The cathode Cu substrates were serrated, whereas the anode Cu substrate was nearly flat, as shown in Fig. S2c and d, which indicates that the cathode was consumed more severely than the anode. Full-Cu_6_Sn_5_ joint formed soon after 60 min and full-Cu_3_Sn joint formed in 360 min, according to Fig. S2c and f.

Keeping the current density constant at 1.0 × 10^2^ A/cm^2^ and increasing the temperature to 300 °C, the growth rate of both Cu_6_Sn_5_ and Cu_3_Sn increased notably. Similar cellular morphology appeared again to anode Cu_6_Sn_5_ grains, which would greatly increase the solid-liquid interfacial surface area for Cu atoms to deposit, as shown in Supplementary Fig. S3. Full-Cu_6_Sn_5_ joint formed after 60 min and full-Cu_3_Sn formed within 360 min, as shown in Fig. S3c and f.

According to the Cu-Sn phase diagram, the liquidus temperature of Cu_6_Sn_5_ is above 227 °C, while the liquidus temperature of Cu_3_Sn is above 415 °C. Therefore, at 260 °C, Cu_6_Sn_5_ is the direct reaction product between Cu and liquid Sn, but the Cu_3_Sn could only be obtained in the solid-state reaction between Cu and Cu_6_Sn_5_. As we know, the diffusion coefficient of Cu in the liquid (${D}_{{Cu}}^{{liquid}}$) is several orders higher than that in the solid (${D}_{{Cu}}^{{solid}}$)^7^. And the equation of electromigration can be expressed as:

1 $${J}_{{Cu}}^{{EM}}={C}\frac{{D}_{Cu}}{{kT}}{z}^{\ast }e{\rho }j$$

where, *C* is the Cu concentration, *T* is the reaction temperature, *k* is the Boltzmann constant, z* is the effective charge of Cu atoms in liquid Sn, *e* is the electron charge, and *ρj* is the product of electrical resistance and current density, representing the electrical field strength. So when the other parameters are constant, the Cu electromigration flux is decided by the diffusion coefficient. Therefore, the electric current can easily influence the Cu diffusion in the liquid rather than in the solid, so the growth of Cu_6_Sn_5_ is more sensitive to electric current than Cu_3_Sn. But the increase of temperature could increase the diffusion coefficient of Cu in liquid and in solid. In summary, increasing electric current density could only accelerate the growth rate of Cu_6_Sn_5_, but the increase of temperature could enhance the growth rate of both Cu_6_Sn_5_ and Cu_3_Sn.

**Figure 3:**
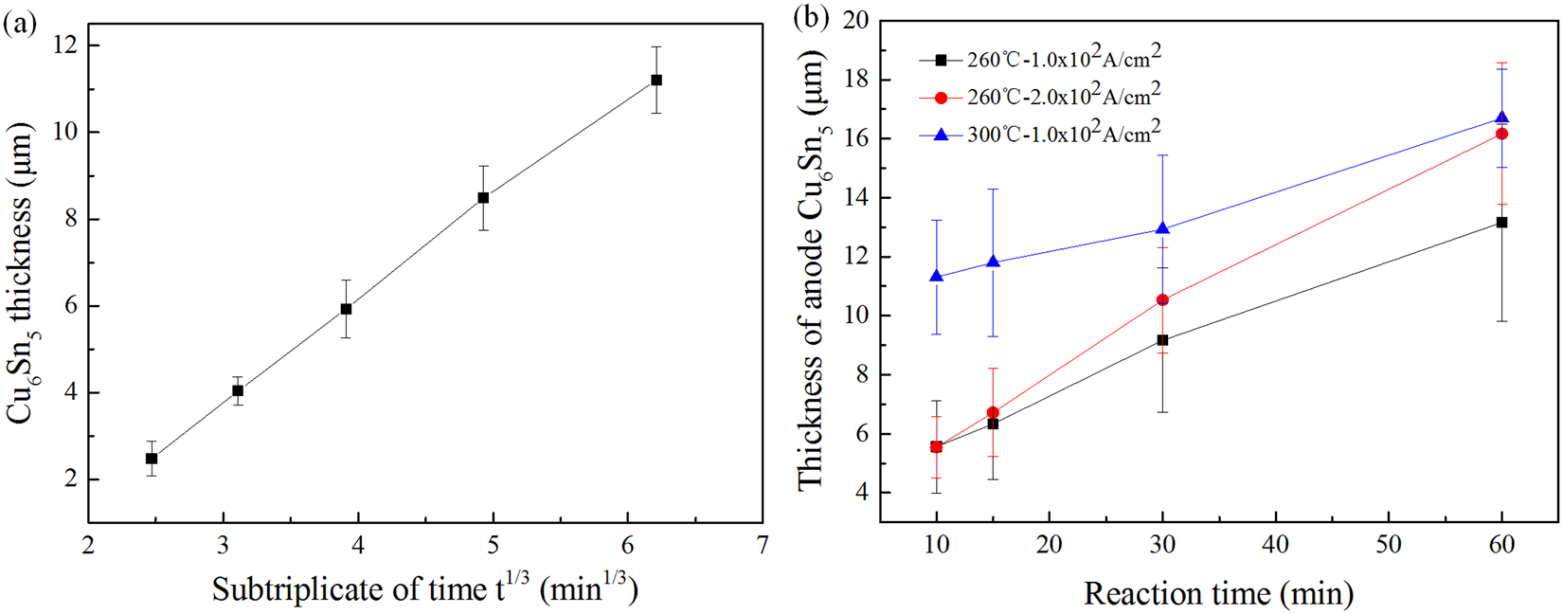
(**a**) The average Cu_6_Sn_5_ thickness at one side vs the subtriplicate of time reflowing at 260 °C; (**b**) The average Cu_6_Sn_5_ thickness at anode side vs time under S-L EM.

Figure 3a shows the change of average Cu_6_Sn_5_ thickness with the subtriplicate of time without electric current applied (the details of thickness calculation are presented in the Supplementary Information). The growth of Cu_6_Sn_5_ followed a t^1/3^ dependence on time *t* under the condition of reflowing, which is the characteristic of supply-controlled ripening process. The relationship was then fitted with a line and the ratio of Cu_6_Sn_5_ thickness to t^1/3^ was calculated as 2.323 μm·s^−1/3^. Figure 3b shows the thickening kinetics of Cu_6_Sn_5_ at the anode in the S-L EM process. It is notable that the Cu_6_Sn_5_ phase grew linearly with time before the liquid Sn was totally consumed, indicating a constant deposition rate at the anode. The IMCs were formed even before reaching the corresponding reflowing temperature because the reactions had already been going on for some time during the heating-up stage. The linear Cu_6_Sn_5_ thickening kinetics can be presented as:

2 $${\rm{\Delta }}{X}_{{C}{{u}}_{{6}}{S}{{n}}_{{5}}}^{{Anode}}={const}\cdot {t}$$

in which the *const* is the ratio of anode Cu_6_Sn_5_ thickness change (${\rm{\Delta }}{X}_{{C}{{u}}_{{6}}{S}{{n}}_{{5}}}^{{Anode}}$) to time (*t*). The value of *const* under three different conditions were then calculated as: 0.107 μm/min under 1.0 × 10^2^ A/cm^2^, 260 °C; 0.212 μm/min under 2.0 × 10^2^ A/cm^2^, 260 °C; and 0.152 μm/min under 1.0 × 10^2^ A/cm^2^, 300 °C. The *const* value nearly doubled when the current density rose to twice the value. Higher temperature could also enhance the growth of anode Cu_6_Sn_5_. The expression of *const* will be then confirmed in the discussion part.

### Top-view microstructure evolutions

**Figure 4:**
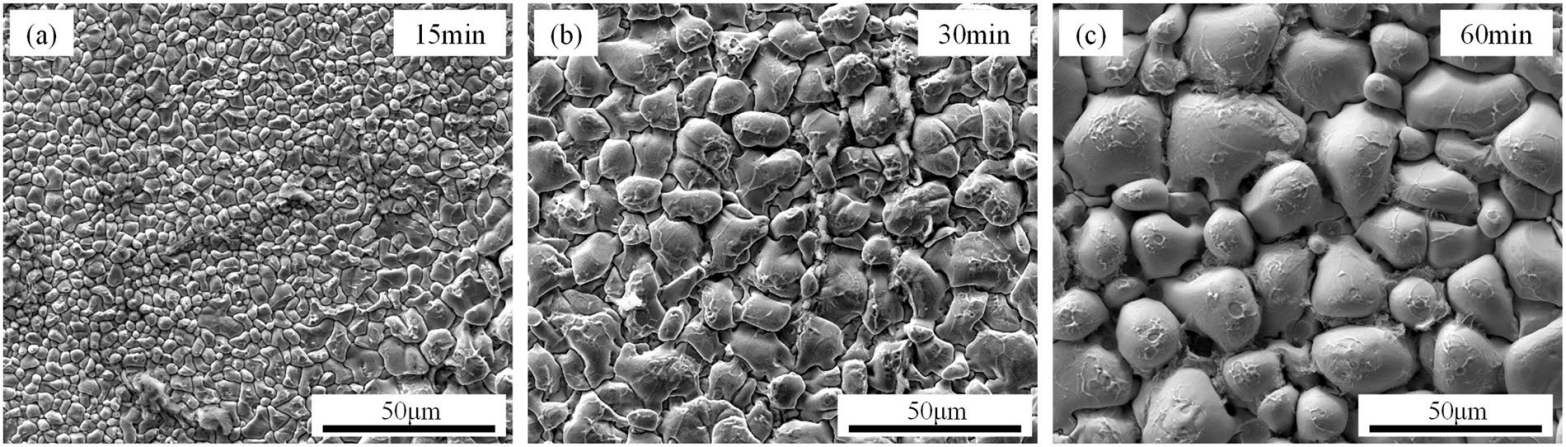
Top-view morphology of Cu_6_Sn_5_ grains reflowing at 260 °C for various time: (**a**) 15 min; (**b**) 30 min; (**c**) 60 min.

**Figure 5:**
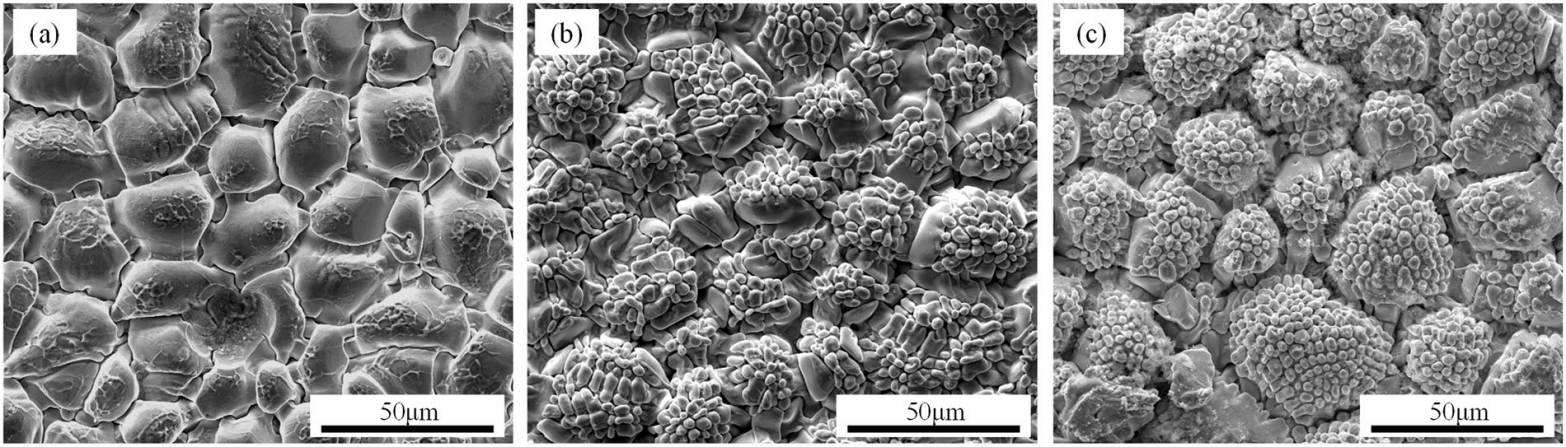
Top-view morphology of anode Cu_6_Sn_5_ grains reflowing under various conditions for 30 min: (**a**) 1.0 × 10^2^ A/cm^2^, 260 °C; (**b**) 2.0 × 10^2^ A/cm^2^, 260 °C; (**c**) 1.0 × 10^2^ A/cm^2^, 300 °C.

Figure 4 shows the three-dimensional morphology of Cu_6_Sn_5_ grains in the joints reflowed at 260 °C for 15 min, 30 min and 60 min. It can be seen that the Cu_6_Sn_5_ scallops coarsened with time. Though there were wrinkles appearing on the surfaces, most of the grains maintained the shape of hemisphere and relatively smooth surfaces. According to the ripening theory proposed by K. N. Tu^17^, if the scallops are assumed to be hemisphere, it follows that the total surface area of the hemispherical scallops of 2πR^2^ is always fixed at twice the base area of πR^2^, in which R represents the diameter of scallops, independent of the size distribution of the hemispheres. It is an important feature for ripening process which has a constant surface area, rather than a constant volume. It is a non-conservative ripening, and the increase in the total volume of Cu_6_Sn_5_ relies on the simultaneous supply of Cu atoms in molten solder. Figure 5 shows the Cu_6_Sn_5_ morphology at 30 min in the other three groups. Figure 5a shows the morphology of Cu_6_Sn_5_ grains reflowed under 1.0 × 10^2^ A/cm^2^ at 260 °C for 30 min, in which more and deeper wrinkles appeared on the surface, showing a tendency of surface area increasing. When the electric current density increased to 2.0 × 10^2^ A/cm^2^ (Fig. 5b), intensive cellular structures formed on the surface, indicating the rule of constant-surface area was broken. With the appearance of this structure, the solid-liquid surface area for reaction was nearly doubled, as the cellular structures were also in the shape of hemisphere. Similar phenomenon happened when the ambient temperature was elevated to 300 °C (Fig. 5c), and the effect of temperature on morphology was larger than that of electric current.

### The mechanical properties of the obtained joints

**Figure 6:**
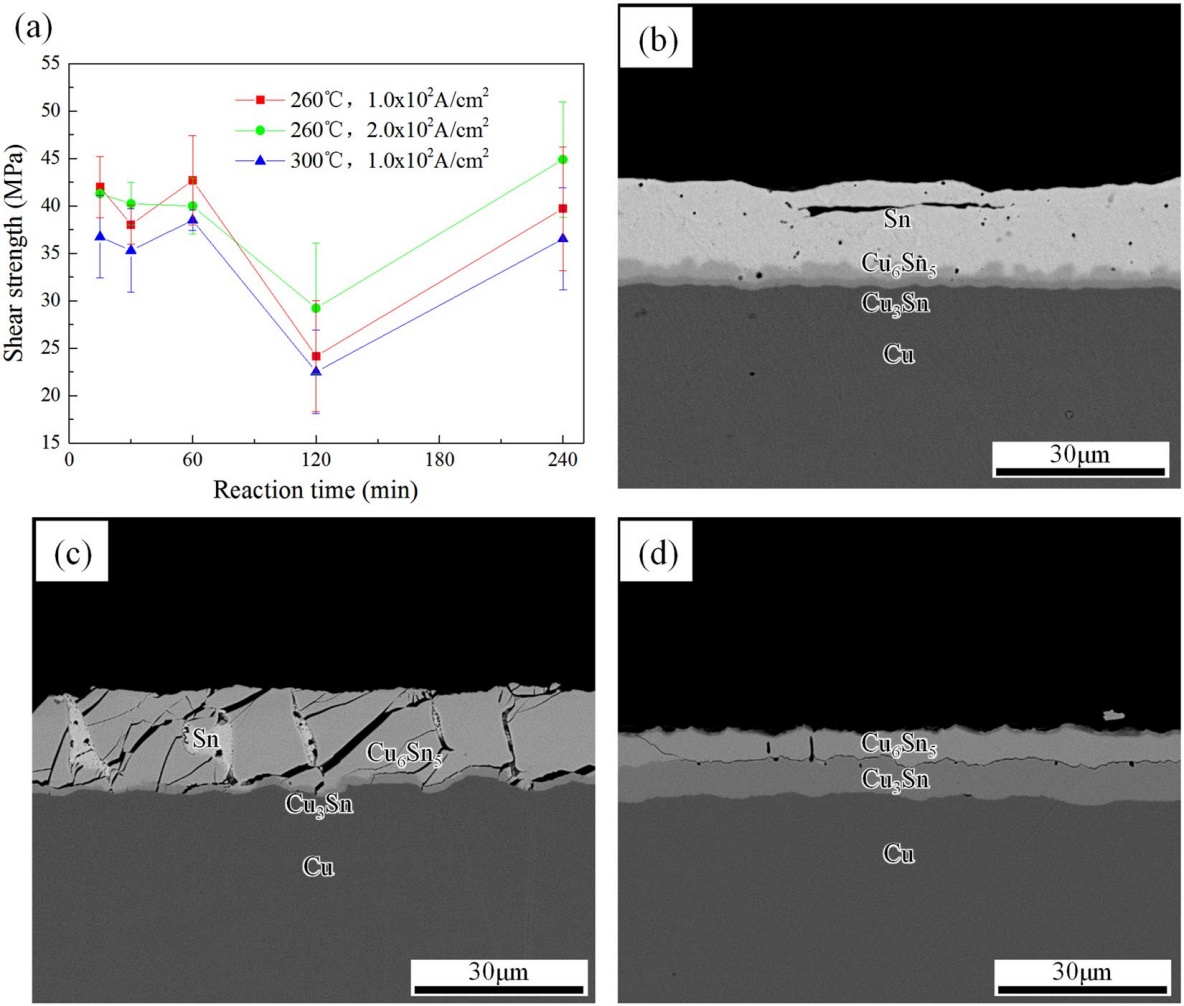
(**a**) The shear strength of the joints as a function of bonding time under different conditions, and SEM images of the corresponding fractures under 260 °C, 1.0 × 10^2^ A/cm^2^ for (**b**) 15 min, (**c**) 120 min, (**d**) 240 min.

Shear tests were conducted to evaluate the mechanical properties of the bonded Cu/Sn/Cu joints under different experimental conditions. It can be seen in Fig. 6a that the shear strength were about 40 MPa and kept relatively constant when the reflowing time was less than 60 min. Before 60 min, there was still a lot of Sn in the joints, so the soft Sn phase absorbed most of the stress and deformation. Figure 6b shows the typical cross-sectional fracture morphology at 15 min, indicating that plastic fracture happened within Sn. At 120 min, the shear strength decreased dramatically, as shown in Fig. 6a. Figure 6c shows cross-sectional fracture morphology at 120 min. The Cu_6_Sn_5_ grains at the anode had combined with the cathode Cu_6_Sn_5_ grains, forming a Cu_6_Sn_5_-Sn mixed structure. The Sn phase was divided and constrained by brittle Cu_6_Sn_5_ phase, and the whole joint could not deform integrally. The mechanical mismatching of the two phase may induce stress concentration to the Cu_6_Sn_5_ grains. This kind of stress may lead to the fracture along the weak boundary between anode Cu_3_Sn and Cu_6_Sn_5_. When the joints were full of Cu_6_Sn_5_ phase after 240 min, the shear strength increased because the defects within the joints were reduced, and the Sn ran out totally. The fractures also happened at the boundary between anode Cu_3_Sn and Cu_6_Sn_5_, and the joint achieved the highest shear strength of 44.88 MPa. In short, the Cu_6_Sn_5_-based joints were able to achieve comparable shear strengths with Sn-based joints as long as the soft phase and defects in them were removed.

## Discussion

### Growth kinetics of Cu_6_Sn_5_ in ripening process

A lot of researchers have investigated the supply-controlled ripening process of Cu_6_Sn_5_ in the conventional soldering progress, and most of them have proved that its growth follows a t^1/3^ dependence on time *t*^18,19^. This phenomenon can be attributed to the rapid diffusion of Cu atoms through channels between Cu_6_Sn_5_ grains. Current studies have verified the existence of channels between Cu_3_Sn and Cu_6_Sn_5_ grains^20^. The width *δ* of these channels is only about 50 nm, which is much smaller than the average thickness of Cu_6_Sn_5_ layers, *X*_*Mean*_. These channels exposed the fresh surface of Cu substrates and served as rapid diffusion paths for Cu atoms to go into the molten solder, whereas the volume diffusion through IMC layer can be ignored. However, with the coarsening of Cu_6_Sn_5_ grains, the number and the area of channels decrease, and the supply of Cu atoms reduces. That is the reason why the Cu_6_Sn_5_ deposition rate decreases with time rapidly. To reduce the unknown parameters, the distance between the top and the bottom of Cu_6_Sn_5_ scallops is replaced by the average thickness of Cu_6_Sn_5_ layers, *X*_*Mean*_. The linear relationship between them was proved in previous literature^20^. This process could be approximately described by the equation below based on Fick’s first law^17^:

3 $$\frac{\partial {X}_{{Mean}}}{\partial {\rm{t}}}\approx -{D}_{{Cu}}^{{channel}}\frac{{\rm{\Delta }}C}{{X}_{{Mean}}}(\frac{\delta }{{X}_{{Mean}}})\frac{{V}_{{C}{{u}}_{{6}}{S}{{n}}_{{5}}}}{{6}}$$

in which, ${D}_{{Cu}}^{{channel}}$ is the diffusion coefficient through channels, ΔC is the Cu concentration difference between the top and the bottom of Cu_6_Sn_5_ scallops, ${V}_{{{\rm{Cu}}}_{{6}}{{\rm{Sn}}}_{{5}}}$ is the volume of Cu_6_Sn_5_ per mole. Therefore, the average thickness of Cu_6_Sn_5_ has a cubic root dependence on time by integrating equation (3):

4 $${\rm{\Delta }}{X}_{{Mean}}\approx {K}\sqrt[3]{t}$$

in which *K* is the scale factor, ${K}=\sqrt[3]{\frac{1}{2}{D}_{{Cu}}^{{channel}}{\rm{\Delta }}C{\delta }{{V}}_{{C}{{u}}_{{6}}{S}{{n}}_{{5}}}}$. The value of *K* was calculated as 2.323 μm/min using the parameters in the Supplementary Table S1. Assuming that the value of ΔC is similar to the saturation concentration of Cu, the ${D}_{{Cu}}^{{channel}}$ can be calculated as 9.98 × 10^−14^ m^2^/s, which is similar to the diffusion coefficient in grain boundaries.

### Growth kinetics of Cu_6_Sn_5_ in S-L EM

**Figure 7:**
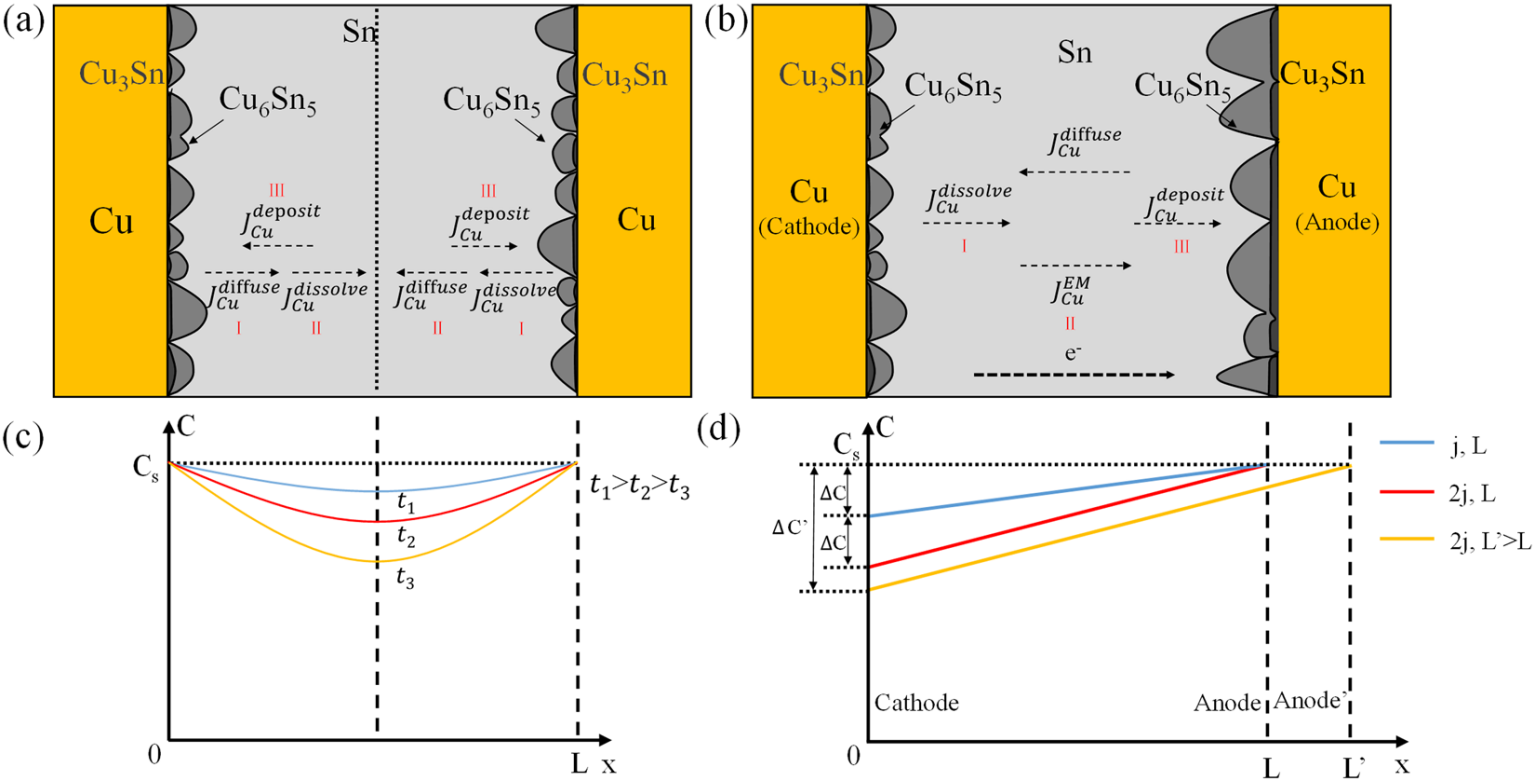
The schematic of Cu flux in molten solder joints under: (**a**) conventional soldering without electric current (**b**) solid-liquid electromigration; The schematic of Cu concentration distribution in molten solder joints under: (**c**) conventional soldering without electric current (**d**) solid-liquid electromigration.

Under the influence of electric current, the growth kinetics of Cu_6_Sn_5_ changes greatly. The electric current changes the distribution of Cu atoms dissolved in molten Sn. In conventional soldering process without electric current, two Cu substrates are the main sources of Cu atoms, as shown in Fig. 7a. Cu concentration distributes systematically in the joint, where the concentration near the substrates is the highest as shown in Fig. 7c. According to Dybkov’s model^21^, the dissolution rate of Cu depends on the concentration in the solid-liquid boundary layer, which can be expressed as:

5 $${J}_{{Cu}}^{{dissolve}}={D}_{{Cu}}^{{liquid}}\frac{{C}_{s}-C}{w}$$

where ${J}_{{Cu}}^{{dissolve}}$ is the Cu flux of dissolution; *w* represents the width of solid-liquid boundary layer, which can be influenced by many external conditions, such as the temperature, convection and so on; C is the Cu concentration and *C*_*s*_ is the solubility of Cu in the molten solder at the reaction temperature. When the molten Sn is gradually saturated by Cu, ΔC decreases with time, and the dissolution rate of Cu substrate slows down. However, when the electric current is applied to the joint, Cu atoms are able to travel across the joint within a few minutes under electromigration, and a Cu concentration gradient from anode to cathode will be established to balance the electromigration flux. At the cathode, the electric current creates a Cu depletion zone where the Cu concentration is always below its solubility. To maintain the solubility equilibrium, the Cu-Sn IMCs at the cathode keep dissolving into the molten solder. Therefore, the Cu_6_Sn_5_ grains at the cathode maintain small size, and the area of channels will not decrease with time. Hence the dissolving rate of cathode will keep constant. At the anode, by contrast, the Cu-Sn IMCs deposit rapidly in high Cu concentration and the area of channels shrinks accordingly, and these channels are ultimately blocked by the rapidly thickening IMCs.

The SEM observations also indicated that the Cu atoms in anode IMCs came mostly from the cathode. By observing Figs 2, S2 and S3, we can notice that the Cu substrate at the anode was flat, and continuous layer-type Cu_3_Sn formed quickly between Cu and Cu_6_Sn_5_ after 15 min, which was a good barrier for Cu diffusion. So the Cu substrate at the anode cannot contact with Cu_6_Sn_5_ and liquid Sn. By contrast, the Cu substrate at the cathode was rather serrated, and the continuous Cu_3_Sn layer did not form until the liquid Sn was totally consumed. Recent study has confirmed that the Cu_3_Sn phase was the reaction product of Cu and Cu_6_Sn_5_ at soldering temperature^22^. So we think most of the Cu atoms diffusing from anode had reacted with Cu_6_Sn_5_, rather than with Sn. Previous researches have also confirmed that during electromigration, the dissolution of cathode was much more serious than the anode^14,23^. So it’s reasonable to assume that the Cu species in anode Cu_6_Sn_5_ came mostly from cathode to simplify the modeling.

So it is obvious that the Cu_6_Sn_5_ growth kinetic model in S-L EM will be greatly influenced by the distribution of Cu atoms in molten Sn. To further confirm the expression of *const* in Eq. (1), the transmission process and the distribution of Cu atoms in the liquid need to be investigated first. There are three main potential rate controlling steps in the growth progress of Cu_6_Sn_5_, as shown in Fig. 7(b): (I) the dissolution rate of cathode Cu substrate; (II) the drift rate of Cu atoms under electromigration in the molten solder; (III) the deposition rate of Cu-Sn IMCs at the anode. The ultimate growth rate is determined by the slowest step, as shown in Fig. 7. The numerical value of deposition rate has already been calculated according to the slope of curves in Fig. 3(a). So the flux of Cu for deposition can be calculated accordingly, as shown in Table 1. However, larger deposition rate of 18.4 × 10^−4^ mol/m^2^·s under 250 °C, 5.0 × 10^2^ A/cm^2^ had been observed in the experiments performed by Haitao Ma^14^, indicating that the upper limit of deposition rate in our study had not been reached. So the deposition cannot be the rate controlling step. The flux caused by electromigration and Cu gradient were expressed as ${J}_{{Cu}}^{{EM}}$ and ${J}_{Cu}^{diffuse}$, respectively. The ${J}_{{Cu}}^{{EM}}$ represents the transmission capability of Cu from cathode to anode. Calculation shows that the drift rate under electromigration is about one order higher than the deposition rate. And the EPMA analysis revealed that the Cu was oversaturated in Sn. So the drift rate cannot be the rate controlling step either. Although the dissolution flux at the cathode is hard to measure by experiments, it must be the rate controlling step. Therefore, the deposition flux of Cu at the anode (${J}_{{Cu}}^{{deposit}}$) is determined by and equals to the dissolution flux at the cathode (${J}_{{Cu}}^{{dissolve}}$). The relationship of the four fluxes can be expressed as:

6 $${J}_{Cu}^{deposit}={J}_{Cu}^{dissolve}={J}_{Cu}^{EM}-{J}_{Cu}^{diffuse}$$

**Table 1:** The values of anode Cu_6_Sn_5_ deposition rate under different conditions.

Number	Electric current density (*j*)/A/cm^2^	Temperature (*T*)/°C	Joint width (*L*)/μm	Deposition rate(${J}_{{\rm{Cu}}}^{{\rm{deposit}}}$)/mol/m^2^·s
1	1.0 × 10^2^	260	30	0.91 × 10^−4^
2	2.0 × 10^2^	260	30	1.79 × 10^−4^
3	1.0 × 10^2^	300	30	1.29 × 10^−4^
Literature^14^	5.0 × 10^2^	250	400	18.4 × 10^−4^

According to the analysis above, a balance equation between deposition and drift flux can be described as below:

7 $${J}_{{Cu}}^{{deposi}{\rm{t}}}=-{D}_{{Cu}}^{{liquid}}\frac{{dC}}{{dx}}+{C}\frac{{D}_{{Cu}}^{{liquid}}}{{kT}}{z}^{\ast }e{\rho }j$$

Given the boundary condition: ${C|}_{x=L}={C}_{s}$, in which *L* represents the average distance between scallop surfaces at both sides. The distribution of Cu in liquid Sn can be confirmed:

8 $$C={C}_{1}+({C}_{s}-{C}_{1}){e}^{\frac{{{\rm{z}}}^{\ast }{\rm{e}}\rho j}{kT}(x-L)}$$

in which ${C}_{1}=\frac{{J}_{anode}kT}{{D}_{Cu}^{{\rm{l}}iquid}{{\rm{z}}}^{\ast }{\rm{e}}\rho j}$. Since the exponential term is small enough compared with *C*, the equation (8) could be simplified as a linear relationship:

9 $$C={C}_{1}+({C}_{s}-{C}_{1})\frac{{{\rm{z}}}^{\ast }{\rm{e}}\rho j}{kT}(x-L)$$

as depicted by Fig. 7d. So the Cu concentration at the cathode can be expressed as:

10 $${C}_{Cu}^{cathode}={C}_{s}\,-\,({C}_{s}-{C}_{1})\frac{{{\rm{z}}}^{\ast }{\rm{e}}\rho {\rm{j}}{\rm{L}}}{kT}$$

which means the cathode Cu concentration is always below the saturation concentration, and the concentration difference of them can be expressed as:

11 $${\rm{\Delta }}C={C}_{s}-{C}_{Cu}^{cathode}=({C}_{s}-{C}_{1})\frac{{{\rm{z}}}^{\ast }{\rm{e}}\rho {\rm{j}}{\rm{L}}}{kT}$$

which increases linearly with the increase of electric current density. Therefore, according to the equations (5) and (6), the dissolution flux and *const* can be expressed as:

12 $${J}_{Cu}^{dissolve}={D}_{Cu}^{liquid}\frac{{C}_{s}-C}{w}\frac{{{\rm{z}}}^{\ast }{\rm{e}}\rho jL}{kT}$$

13 $$const={J}_{Cu}^{dissolve}\frac{{V}_{C{u}_{6}S{n}_{5}}}{6}$$

Calculations has proved that the value of *const* is approximately on the same order of magnitude as the experimental results. At the initial stage, the anode IMCs are thin compared with the width of joints, so the dissolution flux keeps relatively constant under equilibrium state. When the electric current density doubles, the flux of dissolution become twice (comparing the blue line and red line in Fig. 7d), and the cathode Cu substrate depletes much more seriously. Besides, according to previous research by M. L. Huang^15^, the size of solder bump will influence the concentration distribution of Cu atoms in molten solder, and influence the growth rate of Cu_6_Sn_5_. The model raised in our study also exhibits such size effect. By comparing the red line and yellow line in Fig. 7d, it can be seen that when the width L increases to L’ under the same current density of *2j*, the Cu concentration difference at cathode increases from 2Δ*C* to Δ*C*′. So the dissolution flux and the growth rate of anode IMCs will increase accordingly. Therefore, this model can explain why the growth rate of Cu_6_Sn_5_ observed by Haitao Ma^14^ is about 67 times larger than that observed in this study under an electric current density only 5 times higher. The main reason is that the joint in ref.^14^ was about 400 μm in width, which was about 13 times larger than that in this study. So the Cu concentration at the cathode was much lower and could accelerate the dissolution. In addition, the increase of temperature will also increase the ${D}_{{Cu}}^{{\rm{l}}{iquid}}\,\,$and *C*_*s*_, and then enhance the dissolution of cathode Cu. When the anode IMCs are thick enough, the channels at the cathode are blocked by large Cu_6_Sn_5_ grains growing from anode. So the growth rate of anode IMCs will slow down.

The formation of cellular morphology on the surface of Cu_6_Sn_5_ grains under elevated electric current density and temperature could be explained by the theory of constitutional supercooling. The constitutional supercooling will result in the morphological instability of the solid-liquid interface and therefore lead to the formation of cellular microstructures. The requirement for forming cellular structures under constitutional supercooling can be expressed as^24^:

14 $$\frac{G}{R} < {m}{C}_{e}({1}-{k}_{0})/{D}_{{Cu}}^{{liquid}}{k}_{0}$$

where *R* is the rate of deposition, *G* is the temperature gradient, *k*_0_ is the solute distribution coefficient, and *m* is the slope of the liquidus. Based on Eq. 14, the increase of solute concentration (*C*_*e*_) or deposition rate (*R*) can both increase the degree of constitutional supercooling. This kind of morphology change reflects the increase of both deposition rate and dissolution rate.

## Conclusions

The morphology evolution and mechanical properties of Cu_6_Sn_5_ intermetallic compounds under the applied current density of 1.0 × 10^2^ A/cm^2^ and 2.0 × 10^2^ A/cm^2^ at 260 °C and 300 °C in Cu/molten Sn/Cu interconnection system were investigated. The following conclusions were conducted:

1. The Cu_6_Sn_5_ grew symmetrically in the joints reflowing without electric current, but showed obvious polarity effects reflowing under S-L EM. By contrast, the growth of Cu_3_Sn was barely influenced by the electric current. Under elevated electric current density and temperature, cellular structures appeared on the surface of Cu_6_Sn_5_ grains after long-time reaction.
2. The growth of Cu_6_Sn_5_ reflowing at 260 °C followed a cubic root dependence on time, and the diffusion coefficient was calculated to be 9.98 × 10^−14^ m^2^/s. Under S-L EM, the cathode Cu_6_Sn_5_ barely grew, while the anode Cu_6_Sn_5_ grew following a linear relationship with time. The growth rate of anode Cu_6_Sn_5_ was 0.107 μm/min under 1.0 × 10^2^ A/cm^2^ at 260 °C, and was doubled to 0.212 μm/min when the electric current density increased to 2.0 × 10^2^ A/cm^2^. Besides, an elevated temperature of 300 °C could increase the growth rate of anode Cu_6_Sn_5_ to 0.152 μm/min under 1 × 10^2^ A/cm^2^.
3. A growth kinetics model of Cu_6_Sn_5_ based on Cu concentration gradient concerning size effect was established to explain the acceleration of anode Cu_6_Sn_5_ growth under electric current. This model proves that higher electric current density, higher temperature and larger joint width can enhance dissolution of cathode Cu, and accelerate the growth of anode Cu_6_Sn_5_. This model can be helpful to predict the growth rate of Cu_6_Sn_5_ in the joints of different sizes under different electric currents at soldering temperatures.
4. The Sn-based joints exhibited plastic fracture features within Sn phase and reached a shear strength of 42.69 MPa. The shear strength of Cu_6_Sn_5_-Sn mixed joints decreased dramatically because the mechanical property mismatching of Cu_6_Sn_5_ and Sn, and the fracture happened mainly along the Cu_6_Sn_5_-Cu_3_Sn boundary. The Cu_6_Sn_5_-based joints exhibited brittle fracture and reached an average shear strength of 44.88 MPa. These results showed that full-Cu_6_Sn_5_ joints could achieve comparable shear strength with Sn-based joints as long as the defects and soft phase were removed.

## Methods

### Sample preparation

The samples used in this study were Cu/Sn/Cu sandwich structure. 1 mm-in-thickness, 99.99% purity Cu plates and 50 μm-in-thickness 99.95% purity Sn foils served as base metal substrates and interlayers, respectively. Both of them were cut into 2.5 × 2.5 mm^2^ pieces. In order to minimize the contact resistance and voids/defects within the joints, the surface oxides and dirt were removed from the substrates and interlayers by slightly polishing for 1 min, and ultrasonic cleaning in acetone for 3 min. Flux was used in between to achieve better wettability.

### Bonding process

The joints were bonded by an electric current-assisted bonding process using a specially-designed fixture, as shown in Supplementary Fig. S5. To ensure the thickness consistency among different samples and good contact between the sample and electrodes, an electrode pressure of 0.08 MPa was applied by two springs and calibrated by a dynamometer. The electric current was supplied by DC power. In Liu’s study^13^, the electric current density was as high as 10^4^ A/cm^2^, completing the reaction in only 30 ms, which was not able to observe the reaction process. In order to slow the growth of Cu-Sn IMC, smaller electric current densities were chosen: 1.0 × 10^2^ A/cm^2^ and 2.0 × 10^2^ A/cm^2^ in this study. After assembly, the whole experimental setup was put into vacuum tube furnace to undergo solid-liquid electromigration at 260 °C and 300 °C with the times from 15 min to 960 min. The temperature at the joint was monitored in real time by thermocouple and maintained constant in the aid of a feedback system to eliminate the joule heating effect. Thus the effect of temperature and electric current density can be investigated separately.

### Characterizations

After bonding, two groups of samples were prepared following the same procedure for cross-sectional characterization and shear tests, respectively. The first group of samples for cross-sectional observation were firstly mounted in acrylic resin and then carefully ground and polished. Afterwards, a Quanta 200FEG scanning electron microscope (SEM) with an energy dispersive X-ray spectroscopy microanalysis (EDS) was used to characterize the microstructure evolution and phase composition. The η-Cu_6_Sn_5_ growing at the Sn side and the ε-Cu_3_Sn growing at the Cu side were of different brightness in SEM images, which can be abstracted by Photoshop software. The IMC thickness thus can be calculated. EPMA (EPMA-1600 Electron probe micro-analyzer) was used to measure the Cu concentration in Cu-Sn joints. Another group of samples were prepared to measure the shear strength using an XYZTEC Condor Sigma Bond Tester with a loading rate of 200 μm/s. The fractures were firstly observed using SEM, and then deeply etched by solution (10 vol. % HNO_3_ + 90 vol. % H_2_O) for 5 min to remove the residual Sn for revealing the 3D morphology of Cu_6_Sn_5_ grains.

## Electronic supplementary material

Supplementary Information
