# Supplementary material for: Growth kinetics of Cu6Sn5 intermetallic compound in Cu-liquid Sn interfacial reaction enhanced by electric current
Source: Sci Rep. 2018 Jan 29;8:1775. doi: 10.1038/s41598-018-20100-1 (PMC5788868; doi:10.1038/s41598-018-20100-1)
Supplement: Supplementary file 1 — Supplementary Information [file 41598_2018_20100_MOESM1_ESM.pdf]

Supporting Information

Growth kinetics of Cu_6Sn_5 intermetallic compound in Cu-liquid Sn interfacial reaction enhanced by electric current

Jiayun Feng, Chunjin Hang, Yanhong Tian, Baolei Liu, Chenxi Wang*

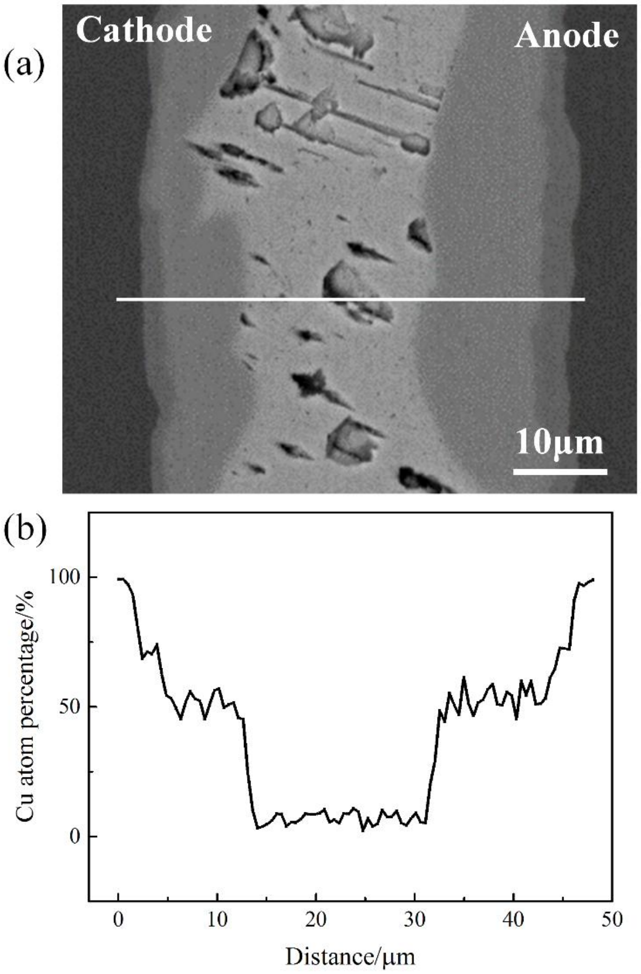

Figure S1. EPMA quantitative line scanning analysis of Cu concentration in Cu-Sn intermetallic joints under a current density of 2.0×10^2 A/cm^2 at 260 °C for 15min.

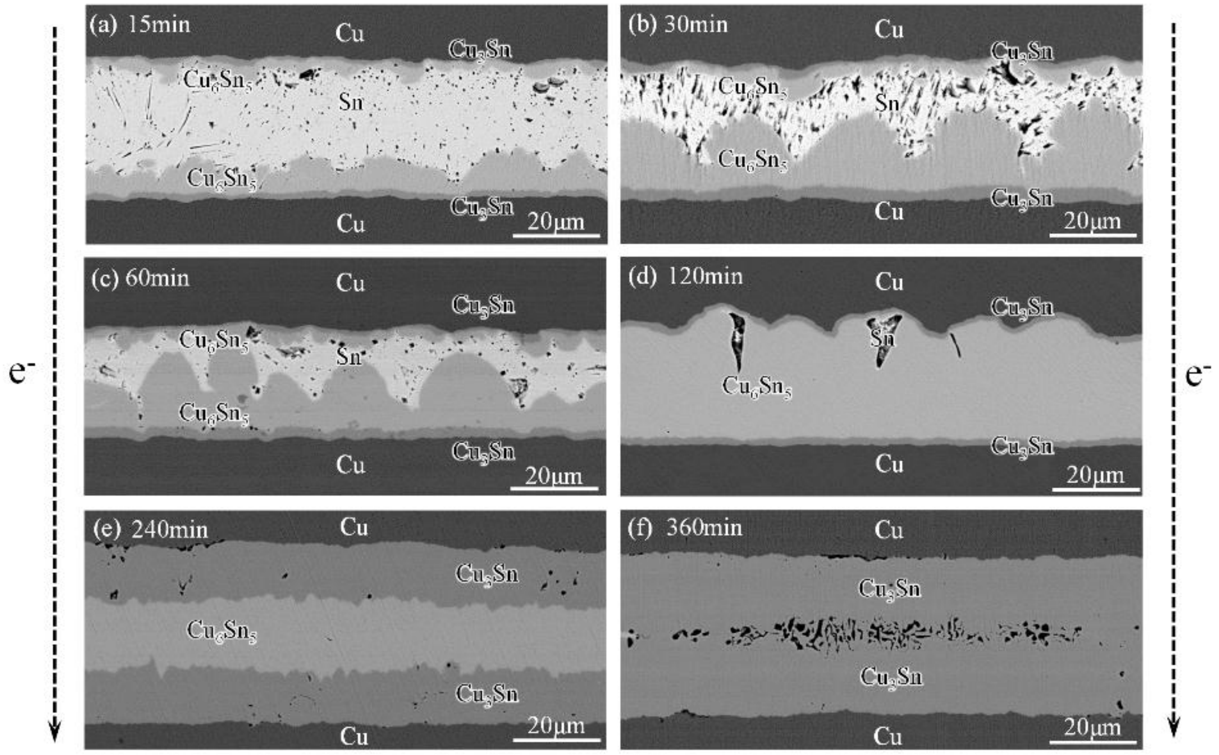

Figure S2. Morphology evolution in Cu-Sn intermetallic joints under a current density of 2.0 × 10^2 A/cm^2 at 260 °C for various times: (a) 15 min, (b) 30 min, (c) 60 min, (d) 120 min, (e) 240 min, (f) 360 min.

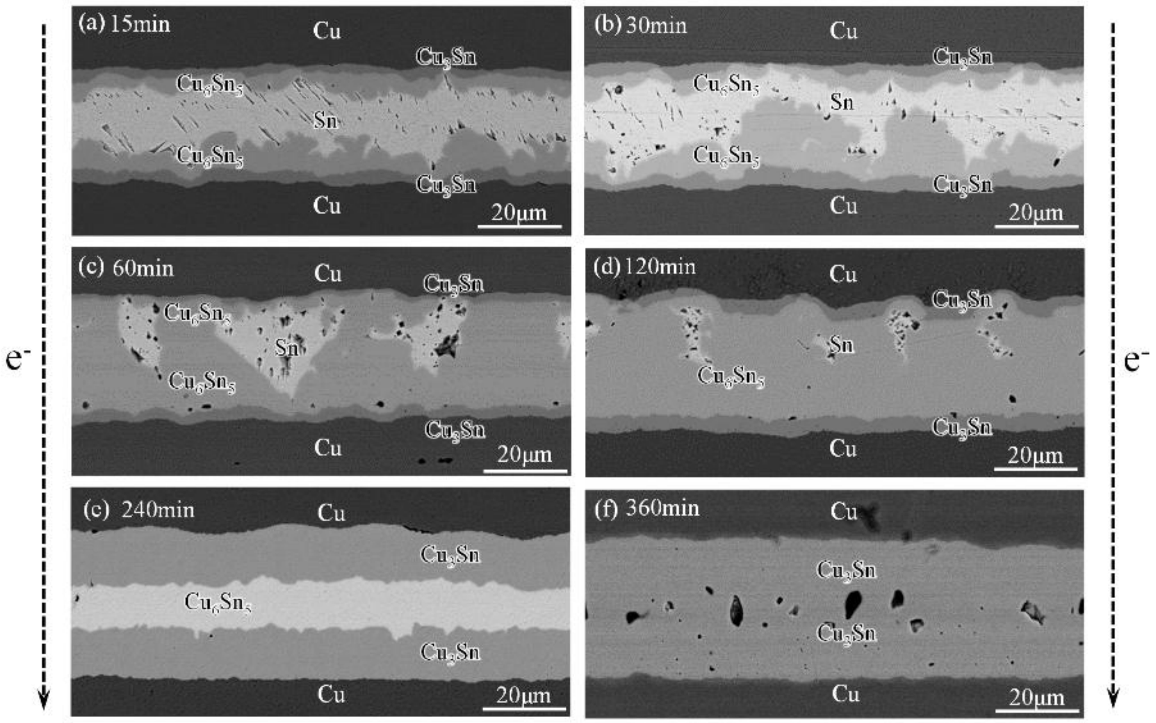

Figure S3. Morphology evolution in Cu-Sn intermetallic joints under a current density of 1.0 × 10^2 A/cm^2 at 300 °C for various times: (a) 15 min, (b) 30 min, (c) 60 min, (d) 120 min, (e) 240 min, (f) 360 min.

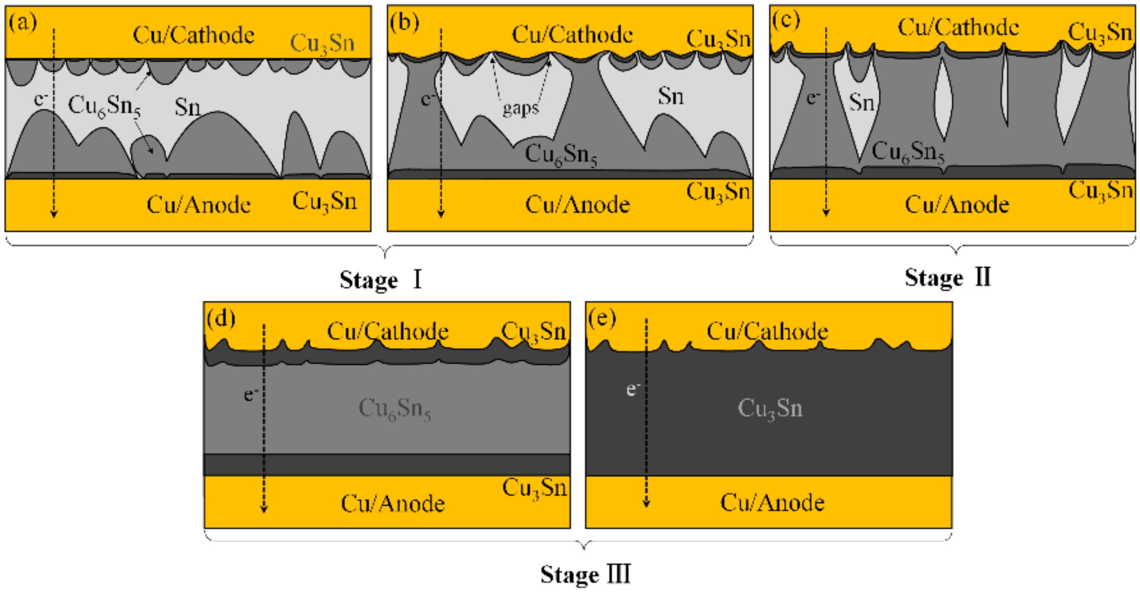

Figure S4. Schematic of Cu-Sn intermetallic morphology evolution with time in solid-liquid electromigration reaction.

Based on the observations of Cu-Sn intermetallic morphology by SEM, the microstructure evolution of Cu-Sn IMCs under the influence of electric current can be depicted by the schematic of Fig. S4. The thickness measurement of Cu_6Sn_5 was mainly divided into the following three stages: In stage Ⅰ, most of the Cu_6Sn_5 scallops were not big enough to touch the other side, and the thickness of Cu_6Sn_5 on both sides were measurable, as shown in Fig. S4 a and b. In stage Ⅱ, most of the Cu_6Sn_5 scallops had touched the other side, and the residual Sn was isolated, so the thickness of Cu_6Sn_5 on either side was nearly indistinguishable, as shown in Fig. S4c. But considering that the cathode Cu_6Sn_5 kept a relatively constant thickness at the early stage, the thickness of Cu_6Sn_5 could also be calculated. The stage Ⅲ is the solid state transformation from Cu_6Sn_5 to Cu_3Sn, as shown in Fig. S4 d and e. Since we only concern about the growth kinetic of Cu_6Sn_5 in solid-liquid reaction, the data of Cu_6Sn_5 thickness was extracted only from the first two stages (before 60 min). For each experimental condition, thickness data from three typical cross-sectional images were measured and averaged.

Table S1. Proximate values of parameters in equation (1) to (14)

Parameters	Values
Mole volume of Cu_6Sn_5 ($\frac{V_{Cu_6Sn_5}}{6}$)	1.97×10^{-5} m^3/mol
Grain boundary diffusion coefficient ($D_{Cu}^{boundary}$)	1.2×10^{-13} m^2/s
Grain boundary size (δ)	50×10^{-9} m
Cu saturation concentration in Sn (C_e)	~1.3 wt.%
Diffusion coefficent of Cu in liquid Sn (D_{Cu}^{liuid})	＞6.44×10^{-9} m^2/s
Effective charge number (z^*)	~2
Electron charge (e)	1.6×10^{-19} C
Electrical conductivity of Sn (ρ)	55×10^{-8} Ωm
Boltzmann constat (k)	1.38×10^{-23} J/k
Electric current density (j)	1.0~2.0 × 10^6 A/m^2

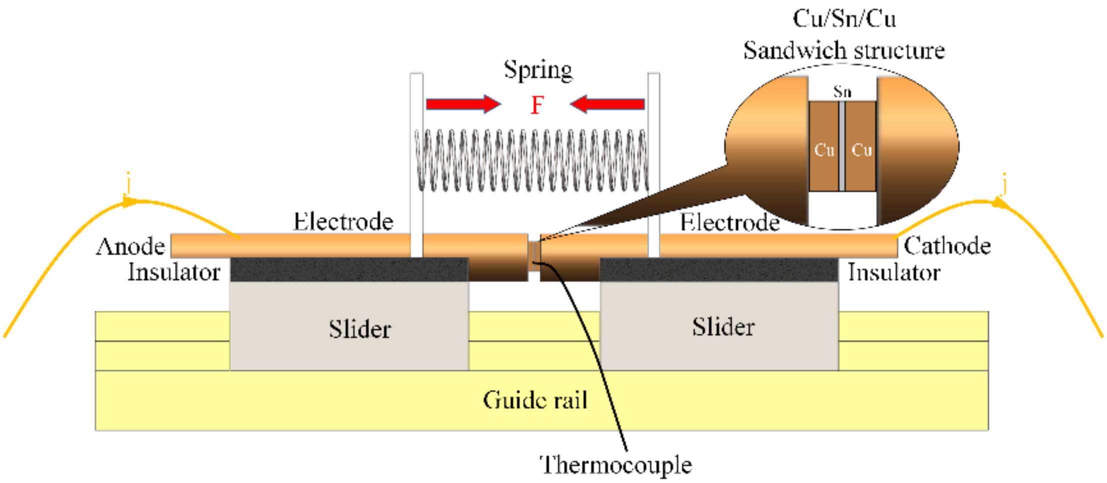

Figure S5. Schematic of experimental set-up for solid-liquid electromigration.
